# Letter to the editor: “The underestimated burden of placental schistosomiasis in endemic regions: Findings from a cross-sectional, diagnostic proof-of-concept study on placental schistosomiasis in Gabon”

**DOI:** 10.1371/journal.pntd.0014819

**Published:** 2026-10-29

**Authors:** Carlotta Helbig, Tina Bohnes, Katja Schneider, Michael Baier

**Affiliations:** 1 Institute of Infectious Diseases and Infection Control, Jena University Hospital, Friedrich Schiller University, Jena, Germany; 2 Institute of Tropical Medicine, University Hospital Tübingen, Tübingen, Germany; 3 Deutsche Gesellschaft für Internationale Zusammenarbeit (GIZ) GmbH, Bonn, Germany; 4 Institute of Medical Microbiology, Jena University Hospital, Friedrich Schiller University, Jena, Germany; University of Oxford, UNITED KINGDOM OF GREAT BRITAIN AND NORTHERN IRELAND

Schistosomiasis remains a Neglected Tropical Disease (NTD) causing a relevant burden in endemic countries [1]. It is also primarily endemic in sub-Saharan Africa, including Gabon, where the predominant species found is *S. haematobium*. Pregnant women represent a significant at-risk population as untreated infection can lead to adverse birth outcomes (ABO) [2]. Therefore, we read the article “*The underestimated burden of placental schistosomiasis in endemic regions: Findings from a cross-sectional, diagnostic proof-of-concept study on placental schistosomiasis in Gabon*” with great interest [3].

The authors screened pregnant women in Lambaréné (Gabon) for *S. haematobium* infection via light microscopy of a single 10 ml urine sample and established a Schistosoma-positive group (n = 28) and a Schistosoma-negative group (n = 20). Placental tissue samples were obtained from all participants and examined for the presence of *S. haematobium* eggs via light microscopy of the macerated tissue. Positive findings were confirmed by qPCR.

Among the women with Schistosoma infection, four women (14%; 4/28) tested positive for the presence of *S. haematobium* eggs in the macerated placenta sample. In the non-infected group, one woman (5%; 1/20) also tested positive for placental schistosomiasis (PS). All five positive results were confirmed by qPCR. By applying these prevalences to the entire cohort, the authors estimate a hypothetical PS prevalence of 6.3%. In contrast, much higher prevalences (22.6% and 19%) of placental schistosomiasis have been described in the literature. This discrepancy raises the question of why the prevalence in this cohort has been estimated to be so much lower than in previously published data.

As the authors already stated, microscopic examination of 10 ml urine for *S. haematobium* is the diagnostic approach in endemic regions and has been recommended by World Health Organization (WHO) [4]. However, urine screening on three consecutive days shows a higher diagnostic sensitivity and would lead to more robust and reliable results [5]. Moreover, urine microscopy is limited by the fact that newly acquired infections in their early stages cannot yet be detected, due to the prepatent period of 10–12 weeks [6]. Alternative or complementary approaches such as antigen detection tests or molecular methods may provide improved diagnostic accuracy [7]. One novel option for diagnosing *S. haematobium* infection described in the literature is the Schistoscope, an automated digital microscope using artificial intelligence [8], that has been field-validated in Lambaréné, Gabon and showed overall non-inferior performance to conventional microscopy [9]. Although the specificity was varying across different studies, tools like this could represent a promising option, particularly in low-resource settings, as they are less reliant on trained microscopists. Hamway et al. evaluated the performance of a novel cell-free DNA qPCR assay based on 20 µl of plasma and found it to have higher sensitivity than conventional diagnostic methods, such as urine filtration microscopy. Such approaches, potentially even from fingerprick blood in the future, should be assessed in further studies [10].

The finding that 5% of the Schistosoma-uninfected group had placental *S. haematobium* eggs in the placenta should be interpreted with caution, particularly considering the small sample size (one woman), as it may reflect limitations in diagnostic sensitivity rather than true maternal infection status. While the authors report the number of eggs per 10 ml of urine and the total number of eggs found in the placenta, it would be helpful to also express placental egg counts relative to tissue weight (e.g., per 50 g or 100 g).

Confirmatory qPCR was performed for all microscopically positive macerated placenta samples and showed concordant results, with all samples also testing positive by qPCR. The target comprised the *Dra1* motif – a highly repetitive sequence of the *S. haematobium* complex also covering *S. intercalatum*. What remains unclear is why the authors did not perform qPCR testing on placenta samples that were negative for *S. haematobium* eggs by microscopy of the placenta. Including these samples could have provided a more diagnostically robust and comprehensive assessment, as molecular methods likely offer a generally higher sensitivity than light microscopy, additionally allowing the detection of egg debris generated through maceration that would otherwise remain unnoticed. Consequently, relying solely on egg detection by light microscopy likely underestimates the prevalence of placental *S. haematobium* in this cohort, as microscopy-negative samples were not subsequently assessed by the more sensitive qPCR method.

In the context of adverse birth outcomes associated with placental schistosomiasis, it would be interesting to gather more information on key parameters like birth weight or gestational age. If these two parameters were available retrospectively, variables like Small-for-gestational-age (SGA) could be assessed or included in future meta-analyses, to compare birth outcomes in women with PS and those without [11]. As placental tissue was examined, it would be interesting to state whether data on placental weight was obtained, as it has been associated with fetal growth [12].

## Abbreviations

ABO: adverse birth outcomes
NTD: Neglected Tropical Disease
PS: placental schistosomiasis
SGA: Small-for-gestational-age
WHO: World Health Organization
